# The Influence of Gap Angle on the Transport Characteristics of Split-Gate AlGaN/GaN Heterostructure Field-Effect Transistors

**DOI:** 10.3390/mi17070831

**Published:** 2026-07-11

**Authors:** Ying Kang, Xiaojia Zhang, Guangyuan Jiang, Chen Fu, Zhenfei Hou, Guangyuan Zhang, Caina Luan, Yang Liu

**Affiliations:** 1 Shandong Key Laboratory of Technologies and Systems for Intelligent Construction Equipment, Shandong Jiaotong University, Jinan 250357, China; 2 School of Information Science and Electrical Engineering, Shandong Jiaotong University, Jinan 250357, China; 3 School of Integrated Circuits, Shandong University, Jinan 250100, China

**Keywords:** AlGaN/GaN HFETs, split-gate, transport characteristics, PCF scattering

## Abstract

In this work, split-gate (SG) AlGaN/GaN heterostructure field-effect transistors (HFETs) with different gap angles were fabricated. The effect of the gap angle on the transport characteristics of these SG devices was investigated via measurement and analysis of their direct-current electrical properties. The results show that varying the gap angle significantly influences the channel current and further modulates the turn-off voltage of the devices. Theoretical analysis indicated that a change in gap angle directly alters the length of the gap region and affects the conduction channel effective width (Weff) through geometric effects, thereby modifying the channel current. In addition, the gap angle affects the total amount and distribution of additional polarization charges underneath the gate, which influences the polarization Coulomb field (PCF) scattering intensity and thus modulates the electron mobility of the devices. These findings provide a new direction for the structural optimization of SG AlGaN/GaN HFETs and offer a valuable reference for further improving the performance of SG devices.

## 1. Introduction

Benefiting from GaN’s outstanding properties, AlGaN/GaN heterostructure field-effect transistors (HFETs) possess a high breakdown voltage and large saturated electron drift velocity, making them well suited to high-frequency and high-power applications [1,2,3,4,5,6]. The split-gate (SG) structure was initially developed for AlGaAs/GaAs heterostructure devices and later extended to AlGaN/GaN counterparts [7,8,9]. Early studies mainly utilized the SG structure to construct quantum wires or quantum dots [10,11,12,13], but it has subsequently been explored as a modulation gate, yielding devices with distinctive performance [14,15,16,17]. Compared with standard AlGaN/GaN HFETs, SG devices have a wider voltage modulation range and lower threshold voltage [14]. When used as voltage amplifiers, they are expected to achieve high linear voltage amplification with lower power consumption [14,18,19]. Nevertheless, our understanding of SG AlGaN/GaN HFETs remains incomplete at present. The gap region, unique to SG AlGaN/GaN HFETs, serves as a conduction path between the source and drain regions after the pinch-off of the gated channel, and its geometry is expected to greatly affect carrier transport. However, there remains a lack of systematic investigations into how the geometry of this gap region affects the transport characteristics of SG AlGaN/GaN HFETs.

In this study, SG AlGaN/GaN HFETs with various gap angles were fabricated to tailor the geometry of the gap region. Electrical measurements confirmed that the gap angle significantly modulates the devices’ channel current and turn-off voltage. Further theoretical analysis revealed that varying the gap angle not only alters the physical length of the gap itself but also affects the conduction channel effective width (*W*_eff_) through geometric effects. The gap angle also affects channel electron mobility by influencing polarization Coulomb field (PCF) scattering. These results confirm that tailoring the gap geometry is an effective strategy to control the transport properties and optimize the performance of SG AlGaN/GaN HFETs.

## 2. Experiments

**Figure 1:**
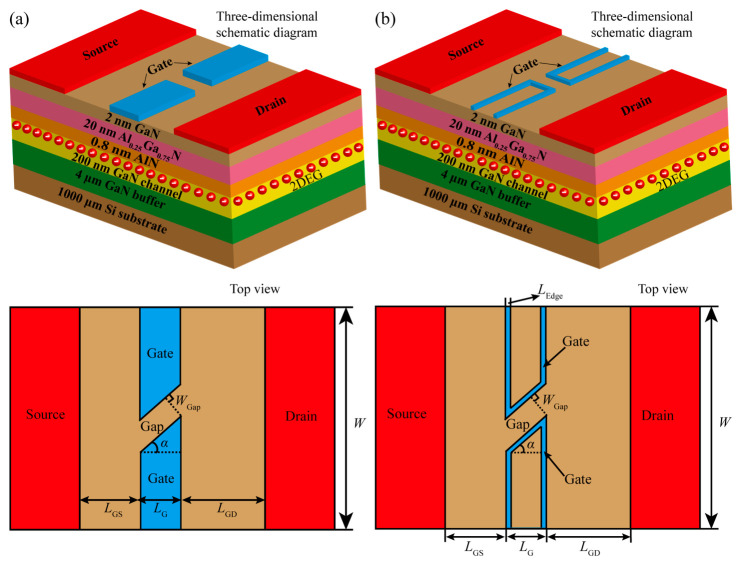
Schematic diagram of (**a**) SG and (**b**) HSG AlGaN/GaN HFETs.

The AlGaN/GaN heterostructure material used in this study was prepared on a 1000 μm Si substrate via MOCVD; its layer configuration is presented in Figure 1. The buffer layer of the material was C-doped to compensate for background carriers and achieve high resistivity, while the channel and barrier layers were unintentionally doped. Hall measurements yielded a two-dimensional electron gas (2DEG) sheet density (*n*_2D_) of roughly 1.06 × 10^13^ cm^−2^ and a Hall mobility of approximately 1936 cm^2^/(V·s). Device isolation was accomplished through fluorine ion implantation. Source and drain contacts were formed by depositing a Ti/Al/Ni/Au (20 nm/130 nm/50 nm/50 nm) stack via electron-beam evaporation followed by rapid thermal annealing at 875 °C for 30 s in a nitrogen atmosphere to ensure ohmic behavior. The specific resistivity of the ohmic contacts was measured to be approximately 4.74 × 10^−4^ Ω·cm^2^ via the transmission line method. The gate Schottky contact was realized by evaporating a Ni/Au (50 nm/150 nm) bilayer, and all device patterning was defined using ultraviolet photolithography during fabrication. Room-temperature electrical characterization was performed using an Agilent B1500A analyzer (Keysight Technologies Inc., Santa Rosa, CA, USA) for current–voltage (I-V) measurements and an Agilent B1520A unit (Keysight Technologies Inc., Santa Rosa, CA, USA) at 1 MHz for capacitance–voltage (C-V) measurements.

Six samples were fabricated and characterized according to the above process. Samples 1–3 are SG devices, the structures of which are shown in Figure 1a, while samples 4–6 are hollow-split-gate (HSG) devices, as illustrated in Figure 1b. In each sample, there is a gap in the middle of the two gates. The dimensional parameters of each sample are listed in detail in Table 1. As can be seen from Figure 1 and Table 1, samples 1 and 4 have identical structures and dimensions, differing only in whether the structure of the gate is hollow. The same relationship applies to samples 2 and 5, as well as samples 3 and 6. Therefore, at the gate pinch-off point, the *W*_eff_ values of samples 1–3 are equal to those of samples 4–6, respectively [20]. In this work, the measurement data for samples 4–6 are used only for the quantitative analysis of *W*_eff_; all other analyses are based on data from samples 1–3.

## 3. Results and Discussion

### 3.1. Analysis of Measured Electrical Characteristics of SG Samples

**Figure 2:**
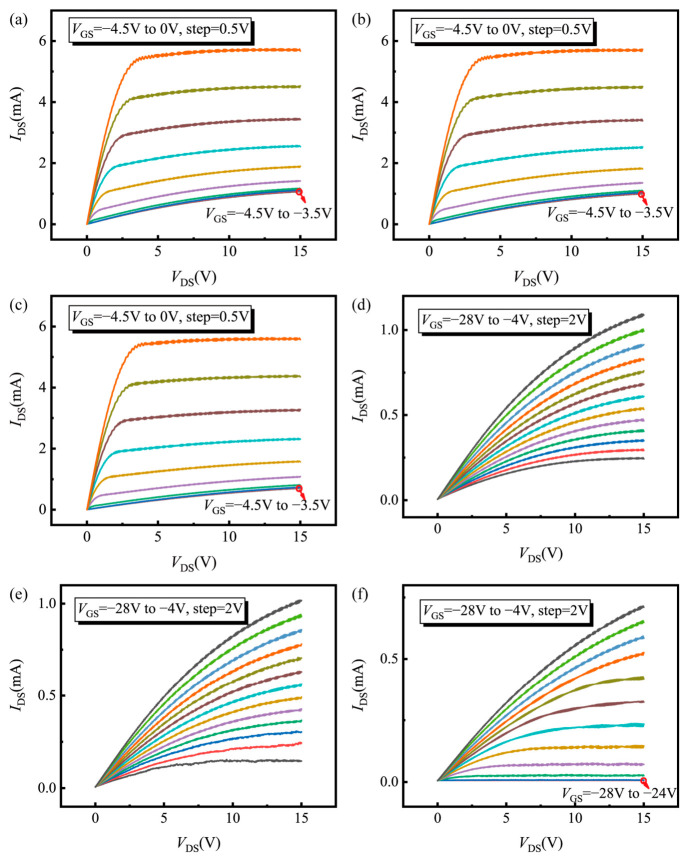
The measured I-V output characteristics of samples (**a**) 1, (**b**) 2, and (**c**) 3 at *V*_GS_ = −4.5 to 0 V and of samples (**d**) 1, (**e**) 2, and (**f**) 3 at *V*_GS_ = −28 to −4 V.

The results of I-V output measurements for samples 1–3 are shown in Figure 2. As can be seen from Figure 2a–c, during the process of reducing the gate bias from 0 to −4.5 V, all three samples exhibited typical characteristics of SG AlGaN/GaN HFETs. Within the gate bias range of −3.5 to 0 V, the drain–source current (*I*_DS_) declines as the gate–source voltage (*V*_GS_) decreases, showing strong gate-controlled modulation. At this time, the channel underneath the gate is not yet pinched off, and the modulation of *n*_2D_ underneath the gate by *V*_GS_ is the dominant mechanism governing the variation in *I*_DS_. When *V*_GS_ is lower than −3.5 V, the channel current is not turned off and can still be modulated by the gate bias, but the modulation capability is significantly weakened. This indicates that the channel underneath the gate is basically pinched off when *V*_GS_ < −3.5 V, and the current is then conducted between the source and drain electrodes through the gap region, with the dominant current modulation mechanism changing to PCF scattering [14]. The gate fringe electric field also contributes to current modulation, yet its influence is weak relative to PCF scattering when *W*_Gap_ is on the micrometer scale. Based on the modulation mechanisms discussed above, the effect of PCF scattering should be strengthened to ensure *V*_GS_ effectively modulates *I*_DS_ after the gated channel is pinched off. Increasing the gate length is a simple and efficient approach to enhance PCF scattering; this justifies the adoption of a large gate length in this work.

**Figure 3:**
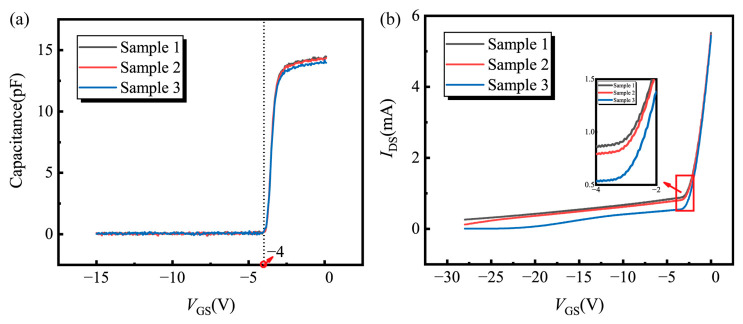
The measured (**a**) C-V and (**b**) transfer characteristics of samples 1–3.

To more accurately extract the gate-channel pinch-off voltage (*V*_P_) for samples 1–3, their C-V curves were measured, with the results shown in Figure 3a. Due to the different gap angles, the gate areas of samples 1–3 are different, leading to differences in their capacitance values. Sample 3 has the smallest gate area and thus the smallest capacitance, while the gate area of sample 1 is slightly larger than that of sample 2, resulting in an insignificant difference in their capacitance values. The *V*_P_ for SG samples can be approximately determined based on the step voltage of the C-V curve [14]. As can be seen from Figure 3a, the capacitances of samples 1–3 decrease rapidly to a minimal value around *V*_GS_ = −4 V, from which the *V*_P_ for the three samples is approximately derived as −4 V. In the above discussion of the I-V output characteristics of samples 1–3, we noted that the gated channel seemed to be pinched off at *V*_GS_ = −3.5 V, which is inconsistent with the *V*_P_ extracted from the C-V curves. This is because when *V*_GS_ = −3.5 V, although the electrons in the gate region are not depleted, *n*_2D_ is much lower than that in the gap region. At this time, the total channel current of the samples is mainly composed of the current in the gap region, and the variation in *n*_2D_ in the gate region, caused by the gate bias, has a negligible effect on this. Therefore, in the *V*_GS_ range of −3.5 to −4 V, the I-V output characteristic curves of samples 1–3 exhibit weak modulation, which is consistent with the characteristics after the pinch-off of the gated channel, leading to an erroneous determination of the *V*_P_. This also indicates that the *V*_P_ extracted from the C-V characteristic curves is more accurate.

To further study the variation in *I*_DS_ with *V*_GS_ after the depletion in electrons underneath the gate, we adjusted the range and step of *V*_GS_ during measurement of the I-V output characteristics; the obtained curves are shown in Figure 2d–f. It can clearly be seen that after the pinch-off of the gated channel, the modulation of *I*_DS_ by *V*_GS_ is still effective despite the extremely weak modulation capability. Comparing Figure 2d–f, it can be observed that sample 3 is essentially turned off at *V*_GS_ = −24 V, while samples 1 and 2 cannot be turned off even when *V*_GS_ is reduced to −28 V. However, although neither of the two samples is turned off, the *I*_DS_ of sample 2 at *V*_GS_ = −28 V is closer to the turn-off state than that of sample 1. This means that, for samples 1–3, a larger gap angle makes it easier to turn off the devices. It should be mentioned that the turn-off state defined in this work differs from the condition where the gated channel is pinched off; it instead refers to the state in which the residual gap-region current is suppressed.

When the drain–source voltage (*V*_DS_) was 10 V, the transfer characteristics of the samples were measured and are plotted in Figure 3b. The transfer curves of samples 1 and 2 are clearly divided into strong- and weak-modulation regions before and after the gate pinch-off point, respectively, while sample 3 exhibits an additional current turn-off region beyond these two regions. All these features are consistent with those observed in the I-V output characteristics. After the channel underneath the gate is pinched off, at a given gate bias, *I*_DS_ decreases as the gap angle increases. The main reason for this trend is straightforward: a larger gap angle directly increases the length of the gap region, raising the overall channel resistance and reducing *I*_DS_. Notably, this trend holds even in some gate bias ranges before the gated channel is pinched off. This behavior is interesting and seemingly counterintuitive. For a sample with a larger gap angle, the gate area is smaller, so fewer 2DEG electrons are consumed under the same negative gate bias. If current flows uniformly parallel from drain to source, devices with less electron consumption should carry larger currents, which contradicts the measured results. This finding indicates that, as electrons underneath the gate are reduced, the 2DEG concentration profile in the channel changes and the electron flow direction is no longer completely parallel to the channel even before the gate pinch-off point, but bends in accordance with the gap angle. This bending lengthens the current path in devices with larger gap angles, resulting in lower current. Although the origins of current bending reported by N. B. Schade et al. may differ from those discussed in this work, they also reported the phenomenon that current propagates along bent paths within straight wire [21]. This may serve as indirect supportive evidence for the above conclusion.

The transconductance (*g*_m_) of each sample can be obtained by differentiating the transfer curve, enabling quantitative comparison of current modulation strength among different samples. However, as shown in Figure 3b, the transfer curves of the three samples are not sufficiently smooth, so direct differentiation fails to yield reliable transconductance values. This non-smooth nature of the measured I-V curves (Figure 2 and Figure 3b) is attributed to the saturation mechanism of SG devices [20,22]. Since the gap region of an SG device is not covered by the gate, the current through the gap cannot saturate via the conventional channel saturation mechanism. Instead, saturation is attained via the virtual gate or device self-heating [22,23,24,25,26]. The velocity saturation originating from self-heating requires an extremely high drain–source electric field, which cannot be achieved within the *V*_DS_ range adopted in this work. On the other hand, the formation of a virtual gate relies on electron trapping by surface trap states, making it difficult to establish a virtual gate with stable potential. Fluctuations in the virtual gate potential consequently induce current variations, which ultimately result in non-smooth I-V characteristics. Introducing an auxiliary gate in the gate–drain region can significantly improve the saturation characteristics of SG devices [22], but such a structure was not used in this work to simplify the theoretical analysis.

As shown in the above analysis, direct differentiation of the transfer curves to calculate transconductance is impractical. To quantitatively compare the current modulation capabilities of the three samples, a fitting method was used to approximate the average transconductance over selected *V*_GS_ ranges. Specifically, linear fitting is performed on the transfer curve in a nearly linear interval, and the slope of the fitted line is taken as the average transconductance for that range. This approach improves accuracy by restricting fits to linear segments. The results are listed in Table 2. Before the channel underneath the gate is pinched off, the transconductances follow *g*_m1_ < *g*_m2_ < *g*_m3_, matching the trend in Figure 3b. Thus, a larger gap angle enhances the gate-controlled modulation in SG samples. The physical origin is clear: within the selected *V*_GS_ range, the transfer curves are approximately linear, so their slopes can be approximately considered to be mainly determined by the current values at the two endpoints. Samples 1–3 share an identical material structure, device geometry, and dimensions, except for the gap angle, so their *I*_DS_ values are very similar at *V*_GS_ = 0 V. As seen in Figure 3b, at *V*_GS_ = −2.5 V, *I*_DS_ is lower for samples with larger gap angles, as explained earlier. Consequently, before the 2DEG electrons underneath the gate are depleted, samples with larger gap angles exhibit higher transconductance in the chosen *V*_GS_ range. After the gate pinch-off point, the transconductances of samples 1–3 show no clear systematic dependence on gap angle. The *I*_DS_ in the gate-free region is governed by multiple factors, primarily the gate fringe electric field, PCF scattering, and *W*_eff_ [14,18,27]. In addition, because the transfer curves in Figure 3b were measured in the saturation region, the surface virtual gate also affects *I*_DS_. Due to the combined influence of these various factors, it is difficult for the transconductance to exhibit regular changes with varying gap angles.

### 3.2. Analysis of the Conduction Channel Effective Width Using HSG Samples

According to our previous work on SG AlGaN/GaN HFETs, after the 2DEG electrons underneath the gate are depleted, the conduction width of the channel in gate–source and gate–drain regions differs from the physical gap width [18,20,27]. To simplify the quantitative analysis, the entire conduction channel is modeled as a rectangular region, whose width is defined as *W*_eff_. For SG AlGaN/GaN HFETs, the structure and size of the devices can affect *W*_eff_ [18,27]. It is difficult to quantitatively determine *W*_eff_ directly via experimental methods; to determine this value requires the use of an HSG structure [20].

**Figure 4:**
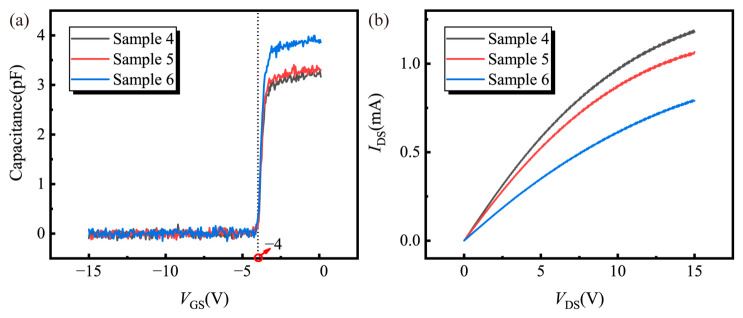
The measured (**a**) C-V characteristics and (**b**) I-V characteristics at *V*_GS_ = −4 V of HSG samples 4–6.

The C-V characteristics of HSG samples 4–6 are plotted in Figure 4a. For HSG samples, a larger gap angle corresponds to a larger gate area, in contrast to SG devices. Accordingly, capacitance increases with the gap angle, as shown in Figure 4a. The gate pinch-off voltage of HSG samples is also approximated from the step voltage in the C-V curve. Since all samples are grown on the same material, their gate pinch-off voltages are theoretically identical, and extraction from the C-V curves confirms this value to be −4 V for all samples. With the gate pinch-off point identified for HSG samples, the I-V characteristics at this bias were obtained, as shown in Figure 4b. After the electrons underneath the gate of the HSG samples are depleted, *I*_DS_ decreases as the gap angle increases, which is similar to what was observed for the SG samples. The most direct cause is the increased gap region length, associated with a larger gap angle. In addition, variations in the gap angle may alter *W*_eff_, which constitutes one of the factors influencing *I*_DS_.

At *V*_GS_ = *V*_P_, the influence of the gate fringe electric field on electrons in the gap region is negligible [7,8,14], so *W*_eff_ can be determined under this condition. For a sample with a gap angle of 0, based on previous research, *W*_eff_ can be expressed as [20]

(1) $$W_{eff}=\frac{L_{DS}}{n_{2D}e\mu_{Total}R_{DS}}.$$

Here, *e* represents the absolute value of electron charge and *μ*_Total_ is the total electron mobility of the conduction channel. For HSG samples, at the gate pinch-off point, *n*_2D_ and *μ*_Total_ in the gate-free region are approximately identical to those of the AlGaN/GaN heterostructure material [20]. *R*_DS_ is the total conduction channel resistance and can be derived from the I-V characteristics of HSG samples under low-field conditions.

For samples with a non-zero gap angle, the physical length of the conduction channel is no longer *L*_DS_, and the length of the equivalent rectangular channel (*L*_eff_) must be adjusted accordingly. The generalized expression for *W*_eff_ can then be rewritten as

(2) $$W_{eff}=\frac{L_{eff}}{n_{2D}e\mu_{Total}R_{DS}}.$$

Here, *L*_eff_ satisfies *L*_eff_ = *L*_GS_ + *L*_GD_ + *L*_G_/cos*α*, where *α* is the gap angle.

Calculations yield *W*_eff_ values of approximately 3.214 μm, 3.113 μm, and 2.845 μm for samples 4–6, respectively. It can be seen that *W*_eff_ decreases as the gap angle increases. Two mechanisms may account for this trend. First, a larger gap angle reduces the number of 2DEG electrons available for conduction in the gate–source and gate–drain regions, increasing the series resistance of these regions and reducing *W*_eff_. Second, a larger gap angle distorts the electron transport path into a more curved trajectory. In contrast with a straight channel, the electron velocity may be altered in a curved channel, increasing overall channel resistance. This resistance rise actually originates from degraded electron mobility, but *μ*_Total_ is assumed to be constant in the derivation of *W*_eff_. The increased channel resistance is therefore reflected as a reduction in *W*_eff_. Based on the present experimental data, it is difficult to distinguish which mechanism dominates or whether both contribute simultaneously. Nevertheless, both mechanisms demonstrate that the geometric shape of the conduction channel affects current transport. This geometric effect will be investigated in detail in future work.

### 3.3. Analysis of Transport Characteristics in SG Samples Based on PCF Scattering

PCF scattering arises from the strong polarization effect in GaN-based materials and is a characteristic scattering mechanism in GaN-based HFETs [28,29,30,31,32,33]. In AlGaN/GaN heterostructure materials, the polarization charge distribution at the interface is almost uniform; thus, PCF scattering is negligible. In AlGaN/GaN HFETs, application of a gate bias renders the polarization charge distribution non-uniform via the inverse piezoelectric effect, inducing PCF scattering [28,34]. For SG AlGaN/GaN HFETs, after the gate pinch-off point, the PCF scattering mainly reflects the interaction between the additional polarization charges beneath the gate and the 2DEG electrons in the conduction channel [14,27]. This interaction is described by the PCF scattering potential, given as [14,27,35]

(3) $$V\left(x,y,z\right)=-\frac{e}{4\pi \varepsilon_{s}\varepsilon_{0}}\iint_{G}\frac{\sigma_{G}}{\sqrt{{\left(x-x’\right)}^{2}+{\left(y-y’\right)}^{2}+z^{2}}}dx’dy’.$$

Here, *ε*_0_ and *ε*_s_ are the dielectric permittivity and static dielectric constant of GaN, respectively. The integration domain G corresponds to the gate region of the SG devices, and *σ*_G_ denotes the additional polarization charge density underneath the gate.

Affected by the PCF scattering potential, electrons in the conduction channel are scattered from an initial state ***k*** to a final state ***k′***. This process is described by the matrix element as [14,27,35,36]

(4) $$\begin{array}{cc} M_{k\to k’} & =A^{-1}\int_{0}^{\infty }\psi_{k’}^{*}\left(z\right)\\ & \times \left[\int_{-\frac{Leff}{2}}^{\frac{Leff}{2}}dx\int_{\frac{W-W_{eff}}{2}}^{\frac{W+W_{eff}}{2}}V\left(x,y,z\right)\exp\left(-iq_{x}x-iq_{y}y\right)dy\right]\psi_{k}\left(z\right)dz\\ & =A^{-1}\int_{0}^{\infty }\psi_{k’}^{*}\left(z\right)\left[V\left(q_{x},q_{y},z\right)\right]\psi_{k}\left(z\right)dz. \end{array}$$

In Equation (4), *A* represents the two-dimensional normalization constant. $\psi \left(z\right)={\left(b^{3}z^{2}/2\right)}^{1/2}\exp\left(-bz/2\right)$ denotes the Fang–Howard variational wave function associated with 2DEG electrons, where $b={\left(33m^{*}e^{2}n_{2D}/8\varepsilon_{0}\varepsilon_{s}\hbar^{2}\right)}^{1/3}$ is the variational parameter, *m*^∗^ is the electron effective mass of the GaN material, and *ℏ* is the reduced Planck constant. Since all electrons involved in the relevant calculations reside within the ungated region, the material’s measured *n*_2D_ is adopted in calculations herein and subsequent derivations. *q_x_* and *q_y_* correspond to the projections of the wave-vector variation ***q*** = ***k*′** − ***k*** along the *x* and *y* axes, respectively. The wave-vector variation ***q*** satisfies $q=\left|2{\left(2m^{*}\hbar^{-2}E\right)}^{1/2}\sin\left(\theta /2\right)\right|$, where *E* is the 2DEG electron energy, and *θ* is the scattering angle of the 2DEG electron from ***k*** to ***k*′**.

Since the actual shape of the conduction channel is unknown, an equivalent rectangular channel with a length of *L*_eff_ and a width of *W*_eff_ is used for approximations in Equation (4). It should be noted that *W*_eff_ is obtained at *V*_GS_ = −4 V, where the channel underneath the gate is just pinched off and the gate fringe electric field is negligible. When *V*_GS_ < −4 V, the fringe electric field effect emerges, which leads to depletion of electrons in the channels on both sides of the gap, thereby narrowing the gap region and further affecting the effective width of the entire channel [14]. Unfortunately, existing methods cannot reliably determine *W*_eff_ with fringe electric field effects included. Also considering the above discussion that the fringe electric field exerts a much weaker impact than PCF scattering, its contribution is neglected in subsequent calculations as a necessary simplification. This approximation would introduce certain errors, especially when *V*_GS_ deviates significantly from −4 V. Since the effect of the fringe electric field is a reduction in *W*_eff_, such a simplification tends to overestimate *W*_eff_ and accordingly yield lower calculated values of *μ*_PCF_. Although quantitative accuracy is limited by the aforementioned errors, the calculation results remain valid for a qualitative comparison among samples 1–3 under identical approximation conditions.

**Figure 5:**
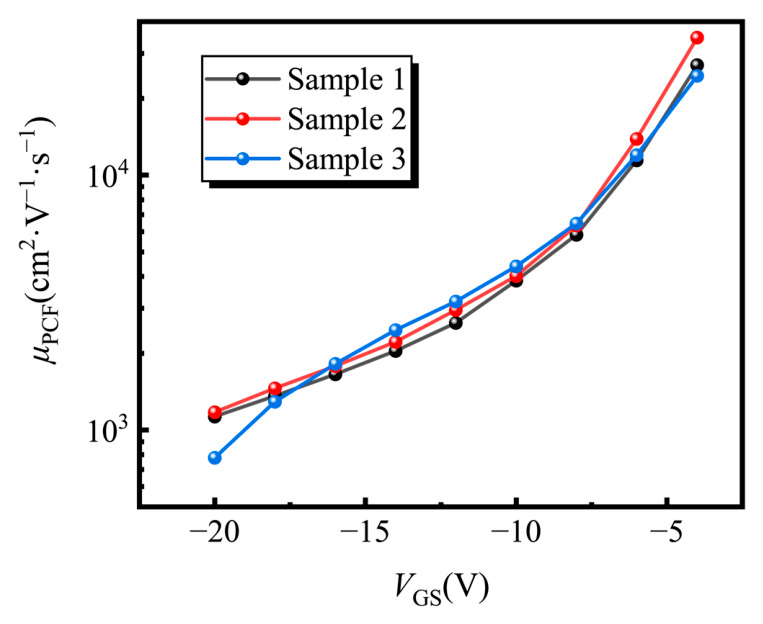
The calculated *μ*_PCF_ for samples 1–3 as a function of *V*_GS_.

Once the matrix element is determined, the derivations concerning PCF scattering can be performed following a standard analytical workflow (Supplementary Section S1) [35,36,37,38,39,40]. The electron mobilities associated with PCF scattering for samples 1–3 at various *V*_GS_ values were computed via a self-consistent iterative procedure [40], and the results are plotted in Figure 5. For all three samples, the mobility decreases with decreasing *V*_GS_. Under negative gate bias, a lower *V*_GS_ corresponds to a larger absolute value of *V*_GS_. Based on the inverse piezoelectric effect, a larger absolute value of *V*_GS_ increases the additional polarization charge density underneath the gate, strengthening PCF scattering and reducing *μ*_PCF_. Comparison of the *μ*_PCF_–*V*_GS_ curves for samples 1–3 reveals no clear systematic dependence of the PCF scattering intensity on gap angle. An isolated comparison of samples 1 and 2 might suggest that *μ*_PCF_ increases with gap angle, but sample 3 breaks this trend. We attempt to explain the origin of this irregular variation in the following.

On the one hand, the gap angle modulates the total amount of additional polarization charges. With all other structural and dimensional parameters fixed, a larger gap angle reduces the total gate area, directly lowering the total amount of additional polarization charges in the gate region. According to Equation (3), the PCF scattering potential decreases accordingly, ultimately leading to an attenuation in PCF scattering and an increase in *μ*_PCF_. On the other hand, the gap angle changes the spatial distribution of additional polarization charges. As the gap angle increases, the entire gap region lengthens; thus, more additional polarization charges accumulate adjacent to both sides of the gap, which lie closest to the gap region. Since PCF scattering is essentially Coulomb scattering, charges closer to the channel electrons produce stronger scattering, as implied by Equation (3). The increased additional polarization charge near the gap thus enhances PCF scattering and reduces *μ*_PCF_. In summary, the gap angle affects both the total amount and the spatial distribution of additional polarization charges, and these two effects have an opposing influence on PCF scattering. When the total-charge effect dominates, *μ*_PCF_ increases with the gap angle; when the distribution effect dominates, the opposite occurs. As a result, the PCF scattering intensity shows no overall regular dependence on gap angle.

## 4. Conclusions

In summary, a series of SG AlGaN/GaN HFETs with different gap angles were fabricated and characterized to explore the influence of the gap angle on their transport characteristics. The I-V characteristics demonstrated that the gap angle affects the turn-off voltage of SG samples, with a larger gap angle corresponding to a higher turn-off voltage. Further analysis of the transfer characteristics showed that the gap angle alters the current flow path even before the pinch-off of the gated channel, thus regulating *I*_DS_. *W*_eff_ values were quantitatively determined using HSG samples, and it was found that *W*_eff_ decreases as the gap angle increases. In addition, the *μ*_PCF_ of SG samples at various *V*_GS_ values was quantitatively calculated under reasonable approximations. It was revealed that the gap angle affects PCF scattering through two competing mechanisms: a larger gap angle reduces the total amount of additional polarization charges and weakens PCF scattering, while it also reshapes the charge distribution to enhance PCF scattering. As a result, *μ*_PCF_ exhibits no regular dependence on the gap angle. This work provides new insights into the structural design and performance optimization of SG AlGaN/GaN HFETs, and the identified geometric effect is worthy of in-depth investigation in future work.

## Figures and Tables

**Table 1:** The dimensions of the six study samples.

	Sample 1	Sample 2	Sample 3	Sample 4	Sample 5	Sample 6
Gate length (*L*_G_, μm)	40	40	40	40	40	40
Gate edge size (*L*_Edge_, μm)	–	–	–	3	3	3
Total channel width (*W*, μm)	100	100	100	100	100	100
Gap width (*W*_Gap_, μm)	3	3	3	3	3	3
Gap angle (*α*, °)	0	30	60	0	30	60
Gate-source distance (*L*_GS_, μm)	5	5	5	5	5	5
Gate-drain distance (*L*_GD_, μm)	5	5	5	5	5	5

**Table 2:** The approximate average values of calculated *g*_m_ for samples 1–3.

	*g*_m_ (S)		
	Sample 1	Sample 2	Sample 3
−2.5 V ≤ *V*_GS_ ≤ 0 V	1.75 × 10^−3^	1.77 × 10^−3^	1.84 × 10^−3^
−20 V ≤ *V*_GS_ ≤ −4 V	2.70 × 10^−5^	2.63 × 10^−5^	2.99 × 10^−5^

## Data Availability

The data that support the findings of this study are available from the corresponding author upon reasonable request.

## Supplementary Materials

The following supporting information can be downloaded at: https://www.mdpi.com/article/10.3390/mi17070831/s1, Section S1: The standard PCF scattering theoretical model and related formulas after determining the matrix element.
