# Supplementary material for: The Influence of Gap Angle on the Transport Characteristics of Split-Gate AlGaN/GaN Heterostructure Field-Effect Transistors
Source: Micromachines (Basel). 2026 Jul 11;17(7):831. doi: 10.3390/mi17070831 (PMC13414170; doi:10.3390/mi17070831)
Supplement: Supplementary file 1 [file micromachines-17-00831-s001.zip › micromachines-4392216-supplementary.pdf]

# The influence of gap angle on the transport characteristics of split-gate AlGaN/GaN heterostructure field-effect transistors

Ying Kang[1,2], Xiaojia Zhang[1,2], Guangyuan Jiang[1,2], Chen Fu[1,2], Zhenfei Hou[1,2], Guangyuan Zhang[1,2], Caina Luan[3], Yang Liu[1,2,*]

[1]Shandong Key Laboratory of Technologies and Systems for Intelligent Construction Equipment, Shandong Jiaotong University, Jinan 250357, China

[2]School of Information Science and Electrical Engineering, Shandong Jiaotong University, Jinan 250357, China

[3]School of Integrated Circuits, Shandong University, Jinan 250100, China

[*]Author to whom correspondence should be addressed: ly2451985210@163.com

## **Supplementary Section S1.** The standard PCF scattering theoretical model and related formulas after determining the matrix element

After determining the matrix element, the energy-dependent scattering rate of PCF scattering can be obtained, and is written as[S1–S4]

$$\frac{1}{\tau_{PCF}(E)}=\frac{Am^*}{2\pi\hbar^3}\int_{-\pi}^{\pi}\left|\frac{M_{k\to k'}}{S(q,T_e)}\right|^2(1-\cos\theta)\,d\theta, \tag{1}$$

where $T_e$ is the 2DEG electron temperature, and $S(q,T_e)$ represents the screening function, which can be expressed by[S1,S2]

$$S(q,T_e)=1+\frac{e^2F(q)\Pi(q,T_e,E)}{2\varepsilon_0\varepsilon_s q}. \tag{2}$$

Here, $F(q)$ is the form factor, written as[S1,S2]

$$F(q)=\int_0^\infty\int_0^\infty \psi^2(z)\psi^2(z')\exp(-q|z-z'|)\,dzdz', \tag{3}$$

and the static polarizability function $\Pi(q,T_e,E)$ can be written as[S1,S2]

$$\Pi(q,T_e,E)=\frac{m^*}{4\pi\hbar^2k_BT_e}\int_0^\infty\frac{1-\Theta(q-2k_F)\left[1-(2k_F/q)^2\right]^{1/2}}{\cosh^2\left[(E_F-E)/2k_BT_e\right]}dE, \tag{4}$$

where $\Theta(x)$ is the usual step function, $k_F=(2\pi n_{2D})^{1/2}$ is the Fermi wave vector, $k_B$ represents the Boltzmann constant, and $E_F$ is the Fermi energy.

Based on the Fermi statistics, the momentum relaxation time of PCF scattering can be obtained as[S2,S4,S5]

$$\tau_{PCF}=\int\tau_{PCF}(E)E\frac{\partial f_0(E)}{\partial E}dE\Big/\int E\frac{\partial f_0(E)}{\partial E}dE, \tag{5}$$

where $f_0(E)$ is the Fermi function, which can be expressed as

$$f_0(E)=\frac{1}{\exp\left[(E-E_F)/k_BT_e\right]+1}. \tag{6}$$

After determining the momentum relaxation time, the electron mobility of PCF scattering can be calculated by[S2,S4,S5]

$$\mu_{PCF}=\frac{e\tau_{PCF}}{m^*}. \tag{7}$$

Throughout the entire calculation process, $\varepsilon_s$ is set to 8.9, and $m^*$ is set to $0.2m_0$, both of which are widely adopted values for GaN materials. Since $\sigma_G$ cannot be accurately determined via theoretical or experimental approaches, the extraction of $\mu_{PCF}$ can only be realized through a self-consistent iterative procedure. The iterative framework fully follows the research work reported by P. Cui et al.[S6]

## References

[S1] C. Luan, Z. Lin, Y. Lv, J. Zhao, Y. Wang, H. Chen, and Z. Wang, "Theoretical model of the polarization Coulomb field scattering in strained AlGaN/AlN/GaN heterostructure field-effect transistors," J. Appl. Phys. **116,** 044507 (2014).
[S2] M. N. Gurusinghe, S. K. Davidsson, and T. G. Andersson, "Two-dimensional electron mobility limitation mechanisms in $Al_xGa_{1-x}N$/GaN heterostructures," Phys. Rev. B **72,** 045316 (2005).
[S3] F. Stern and W. E. Howard, "Properties of Semiconductor Surface Inversion Layers in the Electric Quantum Limit," Phys. Rev. **163,** 816-835 (1967).
[S4] G. Jiang, Y. Lv, Z. Lin, Y. Yang, Y. Liu, S. Guo, and Y. Zhou, "Polarization Coulomb field scattering with the electron systems in AlGaN/GaN heterostructure field-effect transistors," AIP Adv. **10,** 075212 (2020).
[S5] P. Cui, J. Mo, C. Fu, Y. Lv, H. Liu, A. Cheng, C. Luan, Y. Zhou, G. Dai, and Z. Lin, "Effect of Different Gate Lengths on Polarization Coulomb Field Scattering Potential in AlGaN/GaN Heterostructure Field-Effect Transistors," Sci. Rep. **8,** 9036 (2018).
[S6] P. Cui, Z. Lin, C. Fu, Y. Liu, and Y. Lv, "A method to determine electron mobility of the two-dimensional electron gas in AlGaN/GaN heterostructure field-effect transistors," Superlattices Microstruct. **110,** 289-295 (2017).
